# Pathogenic mitochondrial DNA 3243A>G mutation: From genetics to phenotype

**DOI:** 10.3389/fgene.2022.951185

**Published:** 2022-10-06

**Authors:** Danyang Li, Chunmei Liang, Tao Zhang, Jordan Lee Marley, Weiwei Zou, Muqing Lian, Dongmei Ji

**Affiliations:** ^1^ Reproductive Medicine Center, Department of Obstetrics and Gynecology, The First Affiliated Hospital of Anhui Medical University, Hefei, Anhui, China; ^2^ NHC Key Laboratory of Study on Abnormal Gametes and Reproductive Tract (Anhui Medical University), Hefei, Anhui, China; ^3^ Department of Obstetrics and Gynecology, Faculty of Medicine, Prince of Wales Hospital, The Chinese University of Hong Kong, Shatin, Hong Kong, China; ^4^ Wellcome Centre for Mitochondrial Research, Institute of Genetic Medicine, Newcastle University, Newcastle upon Tyne, United Kingdom

**Keywords:** m.3243A>G, phenotype, genetics, heteroplasmy, fertility counseling

## Abstract

The mitochondrial DNA (mtDNA) m.3243A>G mutation is one of the most common pathogenic mtDNA variants, showing complex genetics, pathogenic molecular mechanisms, and phenotypes. In recent years, the prevention of mtDNA-related diseases has trended toward precision medicine strategies, such as preimplantation genetic diagnosis (PGD) and mitochondrial replacement therapy (MRT). These techniques are set to allow the birth of healthy children, but clinical implementation relies on thorough insights into mtDNA genetics. The genotype and phenotype of m.3243A>G vary greatly from mother to offspring, which compromises genetic counseling for the disease. This review is the first to systematically elaborate on the characteristics of the m.3243A>G mutation, from genetics to phenotype and the relationship between them, as well as the related influencing factors and potential strategies for preventing disease. These perceptions will provide clarity for clinicians providing genetic counseling to m.3243A>G patients.

## Introduction

Mitochondria are indispensable organelles that generate around 90% of cellular energy via oxidative phosphorylation (OXPHOS) (Nunnari and Suomalainen, 2012). Human mitochondrial DNA (mtDNA) exists inside mitochondria, and consists of a double-stranded circular DNA molecule of 16,569 base pairs. The mutation rate of mtDNA is 10–100 times higher than that in nuclear DNA (nDNA). Most mutant mtDNA genomes coexist with wild-type genomes in the same cell, a state called heteroplasmy. When levels of pathogenic mutant genomes surpass a threshold of heteroplasmy mtDNA variants can lead to a group of diseases known as mitochondrial DNA-related diseases (Schapira, 2006).

The m.3243A>G mutation was first identified in mitochondrial encephalopathy, lactacidosis, and stroke-like episode (MELAS) syndrome patients in 1990 (Goto, 1990). Found in the mitochondrial *MT-TL1* gene, which encodes mitochondrial tRNA^Leu (UUR)^, it is one of the major pathogenic mtDNA mutations. Its prevalence in Finland was reported to be 0.017% in adult blood (Majamaa et al., 1998), while in Great Britain prevalence was 0.14% in a newborn cohort (Elliott et al., 2008). In Australia, the prevalence was 0.236% in adult hair follicles, which retains the mutation longer than blood (Manwaring et al., 2007), and prevalence ranged from 1.07%–1.69% in diabetes patients’ blood (Masato Odawara and Kamejiro, 1994; Bouhaha, 2010; Wang et al., 2013).

**FIGURE 1:**
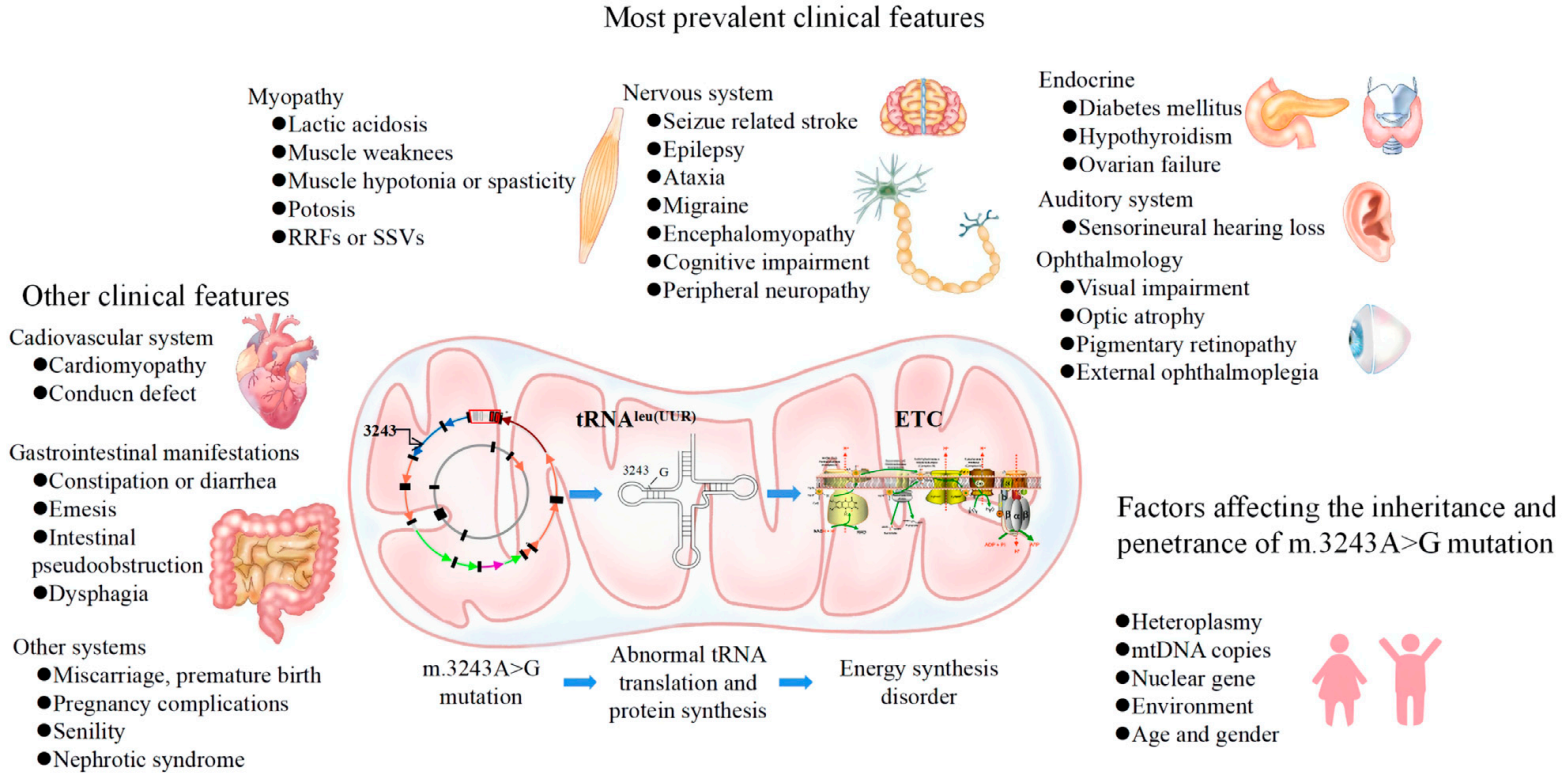
Abstract graph.

The phenotype of m.3243A>G is highly complex and variable, ranging from asymptomatic to lethal phenotypes, partly depending on the level and distribution of m.3243A>G heteroplasmy across cells and tissues (de Laat et al., 2021). There are still no effective treatments for mitochondrial DNA-related diseases; only supportive interventions are available. Therefore, understanding the genetics, molecular mechanisms and phenotypes of the m.3243A>G mutation is important for early clinical recognition, genetic counseling, and enabling the prevention of m.3243A>G inheritance. In this review, we discuss the characteristics of the m.3243A>G mutation, from genetics to phenotypes and the relationship between them, as well as influencing factors and potential strategies for preventing inheritance (Figure 1).

## The genetics of m.3243A>G

### Heteroplasmy dynamics

Heteroplasmy of m.3243A>G can arise *de novo* in somatic cells or be inherited (Stewart and Chinnery, 2021), and levels can be variable across cells and tissues. When somatic cells divide mtDNA molecules are randomly distributed to daughter cells potentially resulting in different heteroplasmy levels between them, a process called vegetative segregation (Stewart and Chinnery, 2015). In non-dividing cells, heteroplasmy is also dynamic due to the process of relaxed replication in which mtDNA is continuously degraded and replicated (Chinnery, 1999). Thus, in both dividing and non-dividing cells, heteroplasmy can drift higher or lower through random genetic drift, or remain steady (Chinnery, 2000).

Even though random drift plays an important role in shifting heteroplasmy in somatic cells, selection has been observed in the case of m.3243A>G. Different tissues may have similar mutant loads at birth but as age increases postmitotic cells, such as skeletal muscle or urine epithelial cells, tend to have higher and more stable heteroplasmy levels than mitotic cells, such as hair follicles and buccal mucosa. The lowest heteroplasmy levels are usually found in blood cells (Shanske et al., 2004).

This phenomenon may be explained by the finding that during the proliferation of mitotic cells, mutant mtDNA can be preferentially selected against and removed, i.e., purifying selection (Burr et al., 2018). It has been shown levels of mutant mtDNA in muscle and blood of MELAS patients can vary (Poulton, 1993), and m.3243A>G mutation load in leukocytes declines by around 1.4%–2.3% per year (Rahman et al., 2001; Grady et al., 2018; Langdahl et al., 2018). Walker et al. found markedly reduced heteroplasmy levels with age in T cells from m.3243A>G carriers (Melissa A. Walker, 2020). Moreover, patients with m.3243A>G were found to carry a lower mutation load in mitotic gastrointestinal epithelial cells compared to smooth muscle cells with ageing (Su et al., 2018). The mechanisms driving such a decline in mutation load are unclear. It is possible mechanisms that enable selective degradation of dysfunctional mitochondria via mitophagy, leading to the removal of mutant mtDNA molecules. Alternatively, cells with a higher mutation load may have a metabolic disadvantage and a shorter lifespan, allowing cells with lower heteroplasmy to dominate.

On the other hand, selection in favour of an mtDNA mutation can occur. Although it seems that selection is less effective in postmitotic tissues, a mechanism may exist to compensate for defective mitochondria by replicating a cell’s entire mtDNA content (Stewart and Chinnery, 2015). While heteroplasmy would nominally remain static, this would expand cells’ OXPHOS capacity. A slight replication advantage has been reported for m.3243A>G carrying genomes due to decreased binding of the mitochondrial termination factor (mTERF) and less replication pausing at this locus (Hyvarinen et al., 2007). In the short term, a mechanism as described above will ensure a sufficient number of wild-type mtDNA. However, m.3243A>G mutation load would increase, eventually compromising the cell. Such a phenomenon is also reported for other harmful variants, wherein mtDNA genomes with replicative advantages are replicated at a higher rate despite OXPHOS being compromised (van den Ameele et al., 2020).

### Inheritance

A genetic bottleneck in the inheritance of mtDNA, wherein only a tiny fraction of total mtDNA copies is transmitted from oogonia to primordial germ cells (PGCs) before being replicated back to around 400,000 copies in mature oocytes, induces rapid intergenerational shifts in heteroplasmy (Chiang et al., 2020). Rebolledo et al. estimated the size of the genetic bottleneck to be 30–35 mtDNA genomes (Rebolledo-Jaramillo et al., 2014). Mathematical models predict that subtle differences in the size of the mtDNA genetic bottleneck will have a dramatic impact on the scale of segregation of heteroplasmy (Wonnapinij et al., 2010). Furthermore, Wilson et al. found that patients harboring the m.8993T>G/C showed more rapid segregation of heteroplasmy levels than those harboring the m.11778G>A, m.8344A>G or m.3243A>G mutations (Wilson et al., 2016). After oocyte fertilization, the total number of mtDNA copies is understood to remain constant until the blastocyst implants in the uterus. Surprisingly, Monnot et al. (2013) observed an increase in mtDNA copies in blastocysts relative to zygotes of m.3243A>G patients. Elevated copy number was weakly positively correlated with increased mutation load, suggesting the existence of a compensation mechanism for OXPHOS defects caused by high levels of m.3243A>G heteroplasmy is active in the embryos of carriers (Monnot et al., 2013). At the level of whole embryos, heteroplasmy varies significantly among embryos, but the average heteroplasmy level across all embryos is very close to that of the mother (Monnot et al., 2011; de Laat et al., 2013).

Three mechanisms might exist to explain this variation during transmission. Currently, it is believed that random genetic drift is the main determinant of inherited mtDNA mutation loads, and the Kimura distribution was used to model the random patterns of mtDNA segregation in inheritance (Wonnapinij et al., 2008). This study confirmed the goodness of fit of the Kimura distribution in a human pedigree dataset, which examined the distribution of heteroplasmy across 82 single primary oocytes derived from a carrier of the m.3243A>G mutation, indicating the random genetic drift. Another study of 577 mother-child pairs transmitting the m.11778G>A, m.3460G>A, m.8344A>G, m.8993T>G/C and m.3243A>G mtDNA mutations also found no evidence of selection during transmission (Wilson et al., 2016).

It remains possible the inheritance of m.3243A>G, and other mtDNA mutations, features selection bias. In mammals, strong purifying selection against some pathogenic mtDNA mutations has been experimentally observed (Burr et al., 2018). Should purifying selection exist in the inheritance of human pathogenic mtDNA mutations, it is unclear where or how. So far it has been shown that in PGCs, wherein the mtDNA bottleneck is understood to take place, a switch from glycolytic metabolism to oxidative metabolism occurs in early development. This coincided with a reduction of non-synonymous mtDNA variants suggesting this may act to expose dysfunctional mtDNA to a selection mechanism (Floros et al., 2018). Ma et al. found that deleterious mtDNA mutations are abundant in mature mouse oocytes and preimplantation embryos of *POLG* mutator females but not in their live offspring, indicating selection might also occur during post-implantation development (Ma et al., 2020).

Meanwhile, Chinnery et al. reported a skewed distribution of m.3243A>G from mothers to offspring suggesting the preferential transmission of mutant genomes (Chinnery, 2000). However, the sample size remains small. Furthermore, the level of m.3243A>G mutation in the blood decreases with age, leading to bias in intergenerational comparisons if unadjusted. Ascertainment bias also likely exists in sampling, as pedigrees with severe or recurring presentations are more likely to be sampled.

Overall, there is no compelling evidence of selection for or against m.3243A>G during inheritance, but further study is necessary. Further work is required but significant technical challenges remain. Evidence suggests selection against pathogenic mutations is linked to follicular atresia (May-Panloup et al., 2016). A high mtDNA mutation burden may also contribute to increased miscarriages in humans (Kaare et al., 2009). Although these possibilities may lead to a sampling bias wherein samples carrying higher levels of mtDNA mutation load are unavailable, definitive evidence is still lacking.

## Pathogenic molecular mechanism

The pathogenesis of m.3243A>G is caused by the perturbed function of the tRNA^Leu (UUR)^, which is primarily responsible for decoding UUR (R = A or G) codons (Yarham et al., 2010). After transcription, tRNAs undergo important post-transcriptional modifications, such as folding into clovers, methylation, and aminoacylation (Richter et al., 2021). The m.3243A>G mutation may affect structural stability, methylation, aminoacylation, or codon recognition of tRNA^Leu (UUR)^, which further affects protein synthesis of ETC components and impairs OXPHOS (Finsterer, 2007). Specifically, the m.3243A>G mutation disrupts the tertiary interaction between the highly conserved base at position 14 in tRNA^Leu (UUR)^ (>90% for adenine) and U8, which is typically involved in an L-shaped tertiary fold (Koga et al., 2012). This leads to faulty tRNA processing and enzyme maturation, resulting in defects in biochemical defects. The m.3243A>G mutation may also lead to hypomethylation of mt-tRNA^Leu (UUR)^, which impairs the function of nuclear-encoded mitochondrial enzymes. A second modification, 2-methylthiolation (ms^2^), was also found to be decreased in peripheral blood cells collected from MELAS patients, although the tRNA^Leu (UUR)^ is not ms^2^ modified itself (Wei FY, 2015). This modification is also reduced on mt-tRNAs decoding Phe, Tyr, Trp, and Ser codons, suggesting feedback regulatory mechanisms adapting the modification of mt-tRNAs in response to aberrant mitochondrial translation. In addition, the m.3243A>G mutation leads to a decrease in leucyl-tRNA synthetase and thus a significant loss in amino-acylation capacity (Park, 2003). There is much evidence that aminoacylation capacities in tRNA^Leu (UUR)^ are reduced due to m.3243A>G, with aminoacylation activity 25 times less efficient *in vitro*, explaining reduced protein synthesis (Koga et al., 2012). Finally, the m.3243A>G mutation obstructs the normal taurine modification of 5-taurino-methyl-2- thio-uridine (taum (5) U) at the first wobble position of tRNA and impairs the pairing of the mt-tRNA^Leu (UUR)^ anticodon with the mRNA codon (Suzuki, 2002).

The resulting energy deficiency stimulates mitochondrial proliferation of small vascular smooth muscle and endothelial cells, leading to vascular lesions and impaired perfusion in the microvascular system of multiple organs (El-Hattab et al., 2015). In addition, the m.3243A>G mutation can also affect nuclear gene expression (Picard et al., 2014), leading to a high glycolysis rate, increased lactose production, and decreased glucose oxidation. Finally, the m.3243A>G mutation shows damaging effects on NADH metabolism, further reducing ATP production, calcium regulation disorders, and increasing cytoplasmic calcium load (McMillan et al., 2019).

## Phenotype

The phenotypes of m.3243A>G are highly variable, with different symptoms in different patients. Symptoms often affect cells and organs with high energy consumption, such as the nervous system, heart and pancreas. Categories of phenotypes include well-defined clinical syndromes, such as MELAS syndrome, myoclonic epilepsy and ragged-red fibers (MERRF) syndrome, and maternally inherited diabetes and deafness (MIDD), or non-syndromic mitochondrial disorders, such as enteromyopathy, hypertrophic cardiomyopathy, and cluster headaches (Shen and Du, 2021). The syndromic phenotypic spectrum of m.3243A>G reported by the UK MRC showed that 10% of patients exhibited MELAS syndrome, 30% MIDD, and 6% MELAS/MIDD, 13% other syndromes and 28% a panoply of non-syndromic clinical features (Nesbitt et al., 2013). Non-syndromic phenotypes reported that hearing loss and diabetes were the most frequent clinical features, followed by stroke-like episodes (Mancuso et al., 2014). Another cohort study involving 136 m.3243A>G MIDD patients observed the following non-syndromic phenotypes: family histories (84.51%), hearing loss (85.71%), central nervous system diseases (29.19%), myopathy (22.98%), oculopathy (23.60%), cardiac disease (23.60%), nephropathy (13.66%) and underweight (41.58%). Different groups were included in these studies and different levels of heteroplasmy were reported across various tissues of those studied, demonstrating the variable phenotypic spectrum of m.3243A>G. In a Chinese cohort, the age of onset of m.3243A>G symptoms was 8.90 ± 5.78 years old, with a male to female ratio of 1.35:1 (Ma et al., 2010). Seizures (76%) and short stature (73%) were found to be the most common symptoms in the second cohort of Chinese pediatric patients (Xia et al., 2016) (Table 1).

**TABLE 1:** The results of recent large cohorts relating m.3243A>G mutation.

Cohort	Heteroplasmy (mean)	Results	References
180 of m.3243A>G patients	Urine (55%), leukocytes (20%)	Blood pressure (BP) was 9 mmHg higher in female patients. Higher BP was a predictor for left ventricular hypertrophy.	Viering et al. (2022)
262 children patients including 138 of m.3243A>G	Leukocytes (66.1%), urothelium (97%)	Phenotype spectrum: MELAS (110, 84.0%); MM (13, 9.9%); MILS (4, 3.1%); MIDD (1, 0.8%); MMC (1, 0.8%); MELAS/LS (1, 0.8%); MELAS/MIDD (1, 0.8%).	Shi et al. (2022)
111 patients with SLE including 66 of m.3243A>G	—	BMI, age, heteroplasmy, sensorineural hearing loss and serum lactate were prediction factors of the risk of SLE.	Ng et al. (2022)
136 of m.3243A>G MIDD patients	Myocardium (67.50%), blood (26.97%), urothelium (58.13%), muscle (49.53%), hair follicle (41.35%), buccal mucosa (48.25%), nail (32.00%)	Family histories (84.51%), hearing loss (85.71%), central nervous system diseases (29.19%), myopathy (22.98%), oculopathy (23.60%), cardiac disease (23.60%), nephropathy (13.66%), underweight (41.58%).	Yang et al. (2021)
151 of m.3243A>G carriers	Buccal saliva (36%), leucocytes (14%), Urothelium (51%)	m.3243A>G mutation causes a slowly progressive disease, the clinical phenotype being the only determinant of disease progression.	de Laat et al. (2021)
116 patients including 41 of m.3243A>G	—	55% of all participants reported headache and more common in females. Severe impact of migraine on health-related quality of life.	Tiehuis et al. (2020)
267 patients including 65 of m.3243A>G	—	Primary cause of death was cardiovascular disease in 16 patients (26.2%), respiratory in 11 (18.0%), and gastrointestinal in 5 (8.1%). Diabetes, intraventricular cardiac conduction defects and focal brain involvement were independent predictors of death.	Papadopoulos et al. (2020)
32 adolescents with MELAS carrying m.3243A>G	60.4% ± 18.4% (range: 22.5‒100)	The mutational load of subjects inversely correlated with first symptom onset, age at diagnosis of MELAS syndrome, and DM.	Chae et al. (2020)
17 participants carrying the m.3243A>G and 17 healthy controls	Leukocytes (16.3% ± 2.7%), saliva (22.7% ± 3.4%), urine (57.1% ± 6.1%)	Higher heteroplasmy levels in peripheral blood leukocytes were associated with increased levels of glycated albumin and HbA1c, and decreased total hip bone mineral density T-score after adjustment for age and sex.	Geng et al. (2019)
139 live births including 62 carrying m.3243A>G	—	Women who carried the m.3243A>G mutation appeared to be at higher risk of complications during pregnancies, caesarean section and preterm delivery than the unaffected women or those with other forms of mitochondrial disease.	Feeney et al. (2019)
789 elderly men and women	Ranged from 0% to 19% in leukocytes	Elevated heteroplasmy was associated with reduced strength, cognitive, metabolic, and cardiovascular functioning.	Tranah et al. (2018)
238 adult m.3243A>G carriers	Blood (60.1%, age-adjusted)	Age, age-adjusted blood heteroplasmy levels, and sex are poor predictors of phenotypic severity. The presence of nuclear genetic factors influencing clinical outcomes in m.3234A > G-related disease.	Pickett et al. (2018)
102 of m.3243A>G carriers	—	The annual leucocyte mutation level declined by -0.7 (±0.4) percentage points/year, and correlated with the level of the initial sample.	Langdahl et al. (2018)
242 adult m.3243A>G carriers	—	Heteroplasmy, age and mtDNA copy number explain a higher proportion of variability in disease burden, although activity level and disease severity are likely to affect copy number.	Grady et al. (2018)
100 pediatric patients harboring m.3243A>G	Blood (44%)	Half of Chinese pediatric patients with m.3243A>G mutation presented seizures, short stature, abnormal MRI/CT changes, elevated plasma lactate, vomiting, and headache.	Xia et al. (2016)
126 of m.3243A>G carriers	Muscle (61.9% ± 18.6%), blood (27.0% ± 19.9%), urinary (42.9% ± 27.7%)	The high clinical heterogeneity of the m.3243A>G mutation. Hearing loss and diabetes were the most frequent clinical features, followed by stroke-like episodes.	Mancuso et al. (2014)
129 of m.3243A>G carriers	—	10% of MELAS, 30% had MIDD, 6% MELAS/MIDD, 2% MELAS/ CPEO and 5% MIDD/CPEO overlap syndromes. 6% had PEO and other features of mitochondrial disease not consistent with another recognized syndrome.	Nesbitt et al. (2013)
41 of m.3243A>G carriers	Blood (20%), urinary (50%)	Patients with the m.3243A.G mutation have a high incidence of cardiac death and life-threatening adverse events. Left ventricular hypertrophy was the only parameter independently associated with occurrence of these events.	Malfatti (2013)

Note: maternal inherited Leigh syndrome (MILS), maternal myopathy and cardiomyopathy (MMC), mitochondrial myopathy (MM)

### Nervous system

Nervous system symptoms are the most prominent phenotypes of m.3243A>G. 80% of MELAS syndrome cases are associated with m.3243A>G, and the most common age of onset is 2–40 years, although late-onset cases have been reported (Ayman W El-Hattab, 2018). SLEs are a typical symptom of MELAS syndrome, which shows increased T2-weighted signal areas involving both cortical and subcortical areas on temporal, occipital and parietal lobes that do not correspond to the classic vascular distribution and demonstrate variable lesion reversibility (Chen et al., 2020; Ng et al., 2021b). A recent study reported mismatched hypometabolism in SLEs (Finsterer et al., 2021), which may be explained by a reduced ability of mitochondria to utilize glucose.

Epilepsy is a common feature of mitochondrial disease, with both systemic and focal epilepsy observed, and with typically insidious onset and protracted course of disease in cases of m.3243A>G. Meanwhile, patients with *POLG*-related MELAS have rapid onset and progression (Ticci et al., 2020). Impaired neuronal energy metabolism and compromised structural and functional integrity of neurons and neural networks could drive m.3243A>G-related nervous system diseases (Klein Gunnewiek et al., 2020).

Other symptoms include polyneuropathy, migraines, and neurodynia. A recent study of 109 MELAS patients found that 90% of MELAS syndrome patients exhibited peripheral neuropathy, of which 65% had the sensory axonal type and 16% had the sensorimotor axonal type and sensorimotor demyelination type (Luigetti et al., 2016). The prevalence of migraine in patients with m.3243A>G is around 48.8%, much higher than that in the general population, and higher in women than in men (Tiehuis et al., 2020). The m.3243A>G mutation may induce migraines by increasing oxidative stress, impairing endothelial shear stress, and disturbing the synthesis of nitric oxide, arginine, and citrulline (Smeitink et al., 2019). Leigh syndrome (subacute necrotizing encephalopathy) and NARP syndrome (neuropathy, ataxia, and retinitis pigmentosa) are also reported (Liu et al., 2020; Bakare et al., 2021).

### Myopathy

Myopathy is another common manifestation of m.3243A>G, presenting as fatiguability, myalgia, exercise intolerance, and lactic acidaemia (Ng et al., 2021a). Red ragged fibers (RRFs) or strongly succinate dehydrogenase-stained vessels (SSVs) are two muscle biopsy pathological markers for mitochondrial DNA-related diseases and often are associated with a higher heteroplasmy level, as is lactic acidosis (Galnares-Olalde et al., 2021). These markers represent the abnormal proliferation of mitochondria in muscle fibers or constituent cells of intermuscular small vessels (Lu et al., 2020). A large proportion of RRFs, revealed by COX staining, and the presence of SSVs can help distinguish m.3243A>G mutation-related diseases from other mitochondrial diseases (Lu et al., 2020). The majority of myopathies are chronic, although m.3243A>G patients suffering acute rhabdomyolysis with severe lactic acidosis have been reported (Ito et al., 2020). m.3243A>G is also associated with isolated mitochondrial myopathy which one report suggests presents with acute respiratory failure (Mahale et al., 2021).

### Endocrine system

MIDD is the most common phenotype involving the endocrine system, and about 85% of cases can be attributed to m.3243A>G (Robinson et al., 2020). MIDD mainly presents as mature-onset maternally inherited diabetes and sensorineural deafness. The mean age of onset of diabetes is 38 years, developing insidiously with the gradual loss of metabolically active pancreatic B cells. Transitions to insulin dependence usually occur rapidly within 2–4.2 years of initial diagnosis (Boggan et al., 2019). Diabetes in MIDD usually presents similarly to type 2 diabetes, but patients commonly have normal body mass index (Robinson et al., 2020). Reduced insulin sensitivity might occur in the earliest phase of m.3243A>G-associated diabetes, while the occurrence of decreased insulin secretion depends on the heteroplasmy level and the progression of diabetes (Langdahl et al., 2019). Mitochondrial dysfunction may be associated with insulin resistance due to increased production of reactive oxygen species (ROS) and inhibited glycolysis, leading to impairment of glucose uptake (Fazakerley et al., 2018). Other endocrine glands affected include the thyroid, parathyroid, pituitary, and gonadal glands, leading to symptoms of hypopituitarism, short stature, Hashimoto thyroiditis, hypoparathyroidism, and ovarian failure (Shen and Du, 2021).

### Cardiovascular system

Pathogenic mtDNA mutations can lead to reduced myocardial glucose uptake, which adds to impaired cardiac energetics (Lindroos et al., 2016). It was shown that sudden adult death syndrome is a frequent occurrence in patients with m.3243A>G (2.4 per 1000 person-years) and likely to be due to widespread respiratory chain deficiency in cardiac muscle (Ng et al., 2016b). Clinical features include hypertrophic or dilated cardiomyopathy and conduction disturbances, of which hypertrophic cardiomyopathy is the most frequent, and conduction disturbances the least (Finsterer and Zarrouk-Mahjoub, 2020). The most frequent conduction defect in m.3243A>G carriers is Wolff-Parkinson White syndrome followed by ventricular ectopic beats (Niedermayr et al., 2018). The presence of cardiomyopathy worsens the prognosis of patients (Malfatti , 2013). Intraventricular conduction block, diabetes, premature ventricular complexes, and left ventricular hypertrophy were independent predictors of major adverse cardiac events (Wahbi et al., 2015). Imai-Okazaki et al. reported that two children who died of cardiomyopathy presented high burdens of mutation in myocardial tissue (80%–90%), suggesting that high-level heteroplasmy may cause acute severe cardiac disease (Imai-Okazaki et al., 2019). Detection of cardiac disease in m.3243A>G carriers has powerful therapeutic and prognostic implications.

### Vision and auditory system

Mitochondria play an important role in cells such as retinal pigment epithelium (RPE) and corneal endothelium. m.3243A>G has been reported to be involved in vision impairment, including pigmentary retinopathy, macular pattern dystrophy, and optic atrophy (Kisilevsky et al., 2020). A recent meta-analysis found a high prevalence of retinopathy (74.4%) and a linear correlation with visual acuity and age for those with m.3243A>G mutation-related mitochondriopathy. The fifth decade of life was identified as the inflection point for vision loss (Coussa et al., 2021). The fundus presentation of retinopathy has been described to range from mild RPE mottling to advanced and diffuse atrophy. Macular dystrophy is thought to be more frequent in MIDD patients, which might reduce metabolism and decrease oxygen consumption in the retina to protect against the development of diabetic retinopathy (Yang et al., 2021). Although many patients with retinopathy are asymptomatic at an early age, some more sensitive methods (e.g., multimodal imaging with fundus autofluorescence and optical coherence tomography) can aid in earlier diagnosis (Coussa et al., 2021).

Hearing impairment is another common clinical manifestation of m.3243A>G. In a study involving 238 cases of m.3243A>G in Great Britain up to 81% of patients had hearing impairment, which was the most frequent symptom (Pickett et al., 2018). The main types of hearing impairment are sensorineural deafness, which is common in MIDD, as previously mentioned, and peripheral vestibular dysfunction (Hougaard et al., 2019). Hearing loss in MIDD, which affects more men than women, often begins gradually, occurs bilaterally, and may become severe over time (Robinson et al., 2020). By late middle age, the prevalence of m3243A>G patients with hearing loss was up to 767/100 000 (Manwaring et al., 2007).

### Gastrointestinal system

Gastrointestinal manifestations caused by m.3243A>G are very common, with gastrointestinal discomfort, including vomiting, diarrhea, and constipation, reported in 76% of mutation patients (Pickett et al., 2018). The pathophysiological mechanisms are unclear but most likely involve myenteric plexus neuropathy and visceral myopathy with COX deficiency in smooth muscle (Gagliardi et al., 2019). Intestinal pseudo-obstruction (IPO) is a rare but serious gastrointestinal complication with a mortality rate of approximately 50%. Among 226 patients harboring m.3243A>G examined in Great Britain, thirty patients (13%) presented acutely with IPO (Ng et al., 2016a), which might contribute to multiple organ deterioration.

### Other systems

The m.3243A>G mutation has been reported to be associated with accelerated ageing and neurodegenerative diseases. Tranah et al. analyzed 794 elderly individuals aged 70–79 and found that m.3243A>G, even with mutation load as low as 0%–19%, was associated with reduced strength, cognition, metabolism, and cardiovascular function (Tranah et al., 2018). In addition, m.3243A>G can also lead to impairment of renal function. Tanaka et al. (2021) reported a case of chronic kidney disease caused by m.3243A>G, and renal biopsy showed nephrosclerosis with interstitial fibrosis and arteriolar hyaline thickening. Furthermore, m.3243A>G was also associated with pregnancy complications, premature delivery, abortion, and other adverse pregnancy outcomes. Feeney et al. found that pregnant women carrying the mutation had a significantly increased risk of gestational diabetes, dyspnea, and hypertension and an increased rate of cesarean section and premature delivery (Feeney et al., 2019).

### Associations between phenotype and heteroplasmy

The m.3243A>G mutation is usually found heteroplasmic. Only one reported case of homoplasy exists in literature, found in a colon cancer sample (Lorenc et al., 2003). Among m.3243A>G patients, those with higher age-adjusted heteroplasmy levels often have a more severe phenotype and disease progression. Due to the severity and progression of the disease varying widely between individuals, the relationships between the phenotype and heteroplasmy level of m.3243A>G are hard to distinguish. Disease phenotype has been shown to correlate better with the m.3243A>G mutation in urine than in tissues because it includes post-mitotic cells exfoliated from the surface of the renal pelvis, ureter, bladder and urethra (Fayssoil et al., 2017)

In clinical research, it has been reported that increased m.3243A>G heteroplasmy levels in leukocytes are associated with increased levels of hemoglobin A1c and decreased total hip bone mineral density T-score (Geng et al., 2019); m.3243A>G heteroplasmy levels in the buccal epithelium are negatively correlated with myocardial glucose uptake (Lindroos et al., 2016), and age-adjusted blood heteroplasmy levels are significantly associated with the risk of SLEs (Fayssoil et al., 2017). However, in longitudinal studies, the associations become weak. de Laat et al. (2021) followed 151 m.3243A>G patients for 6 years and found phenotypes were slowly progressive, and heteroplasmy levels in leucocytes were only weakly correlated with the severity of the disease. A similar result was reported in MELAS, where heteroplasmy was negatively correlated with the onset and diagnosed age of diabetes, but not with the clinical severity or progression (Chae et al., 2020).

There may be several explanations for the above. First, the level and distribution of heteroplasmy can differ among different tissue types. Tissues with high metabolic levels, such as the brain and heart, are difficult to sample in living humans, so heteroplasmy levels of up to 90% in the brain or heart tissue reported in other studies are often undetectable in living blood samples (Motlagh Scholle et al., 2020). A second reason might be inaccuracies caused by changing heteroplasmy with age. Recent studies have shown that the heteroplasmy levels of age-adjusted blood are more strongly correlated with disease burden (Grady et al., 2018), rather than raw blood heteroplasmy levels. Grady et al. proposed a correction formula for the heteroplasmy level in blood: adjusted heteroplasmy level = blood heteroplasmy/0.977 ^(age +12 years)^ and reported that age-adjusted heteroplasmy levels in the blood, age, and sex were accurate predictors of phenotypes (Pickett et al., 2018).

## Other factors affecting the m.3243A>G mutation

For all pathogenic mtDNA variants, phenotypes generally depend on three factors: heteroplasmy, tissue distribution of pathogenic mtDNA, and the onset heteroplasmy threshold (Gorman et al., 2016). In addition to heteroplasmy, other genetic factors, such as mtDNA copy number and nuclear genetic modifiers, influence the inheritance and penetrance of m.3243A>G mutation.

### mtDNA copy number

mtDNA turnover is a dynamic process that is finely regulated by a balance between replication and degradation. mtDNA copy number is directly correlated with energy demands, oxidative stress and mitochondrial membrane potential (Filograna et al., 2021). Recently, studies found that higher heteroplasmy levels correlated with lower mtDNA copy number in the central nervous system of autopsied m.3243A>G patients (Motlagh Scholle et al., 2020), and mtDNA copy number negatively correlated with disease burden or progression in muscle (Grady et al., 2018). This may reflect an increase in copy number to compensate for impaired OXPHOS. Indeed, Herman et al. found that mtDNA copy number was positively correlated with oxygen consumption in m.3243A>G mutant cybrid cells (Herman, 1996). Cells from different individuals or tissues may differ in their ability to compensate for mutations, potentially leading to variance in phenotypes. In advanced and elderly patients, reductions in muscle mtDNA copy numbers are reported (Filograna et al., 2021). Therefore, changes in mtDNA copy number should be considered as an outcome measure for any clinical trial relating to m.3243A>G-related disease.

### Nuclear genetic factors

Around 1100 nuclear-encoded proteins are localized to the mitochondria, including most OXPHOS proteins. Maeda et al. compared disease progression within 2 pairs of monozygotic twins with m.3243A>G. The twin pairs showed highly similar phenotypes and disease progression, suggesting phenotype is largely determined by genetic factors (Maeda et al., 2016). By quantifying genetic effects on phenotypes, Pickett et al. (2018) reported that nuclear genetic background had a large effect on phenotype, beyond that of heteroplasmy and age. In particular psychiatric involvement was strongly influenced by nuclear factors, while cognition, ataxia, migraine, and hearing impairment were moderately influenced.

**FIGURE 2:**
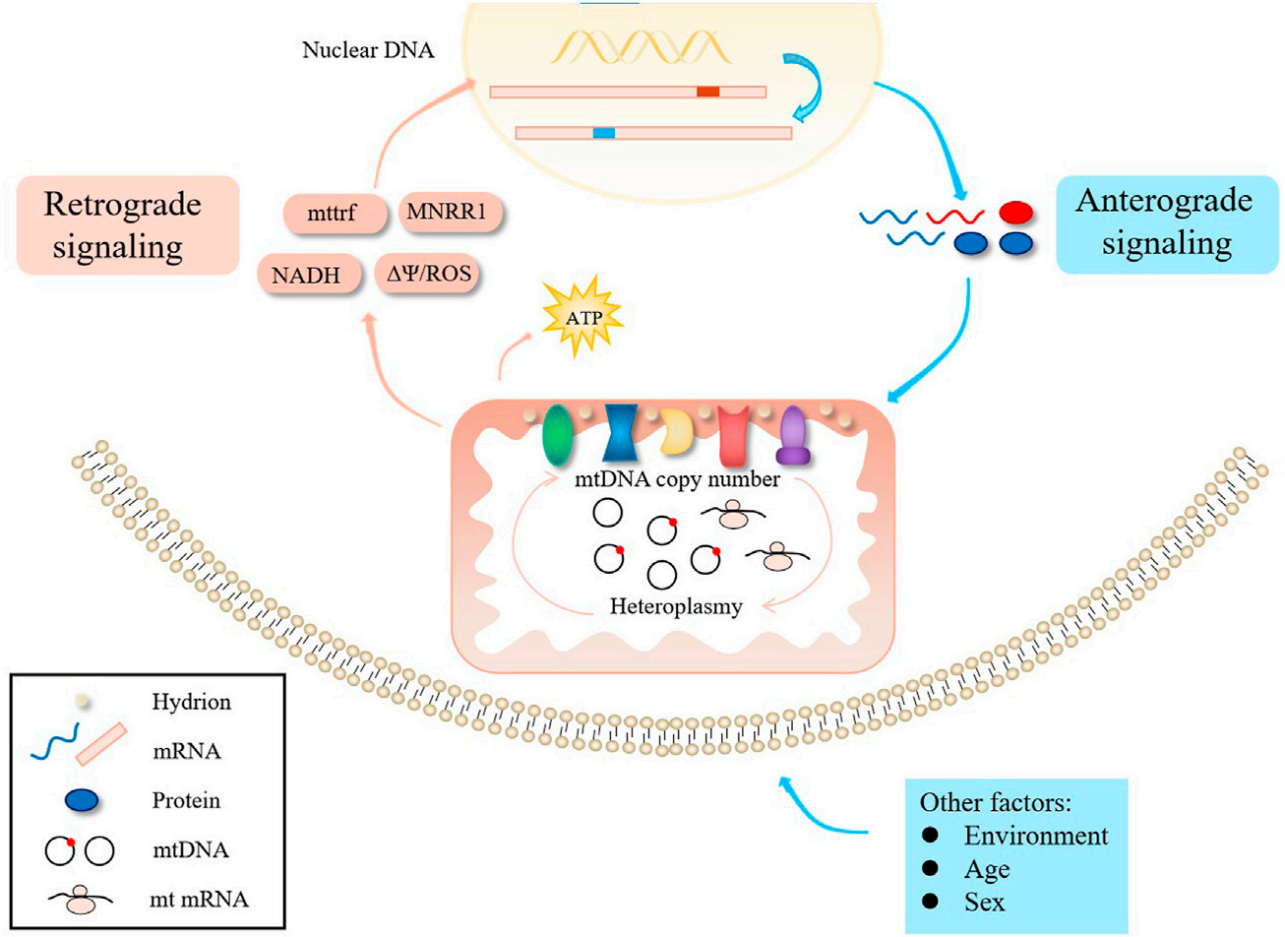
Influencing factors of clinical phenotypes.

The interaction between mitochondria and the nucleus is controlled by anterograde signals in the nucleus, but there is also evidence that retrograde signals mediate the expression of nuclear genes (Figure 2) (Horan et al., 2013). For example, Picard et al. generated somatic cell cybrids harboring variable levels of m.3243A>G and found that small increases in mutant mtDNA caused relatively modest defects in OXPHOS, but resulted in large changes in cellular phenotype and gene expression (Picard et al., 2014). Aras et al. found that increased mitochondrial nuclear retrograde regulator 1 expression can rescue cellular defects caused by m.3243A>G mutation by restoring respiration, decreasing ROS levels, increasing mtDNA copy number, and promoting the mitochondrial unfolded protein response and other protein degradation pathways (Aras et al., 2020). Nuclear genetic factors may thus determine how a cell adapts to the presence of m.3243A>G, explaining some degree of phenotype variation.

### Sex difference

Pickett et al. found sex had a role in determining phenotype among m.3243A>G patients in Great Britain, with the incidence of ptosis, myopathy and stroke-like attacks higher in males (Pickett et al., 2018). A study in Finland also showed that the degree of hearing loss in male patients was higher than that in women, and the male sex was a risk factor for the severity of hearing loss (Uimonen et al., 2001). Males had on average 19.2% higher m.3243A>G heteroplasmy levels in urine, and sex-adjusted urine heteroplasmy levels correlated better with clinical progression (Grady et al., 2018), although this may be due to differences in cell content. Overall, phenotypic differences between sexes may be common in mitochondrial DNA-related diseases.

### Environmental factors

Studies also show that patients with other pathogenic mtDNA variants surpassing critical heteroplasmy thresholds may not present biochemical defects and tissue dysfunction, and this incomplete penetrance may be affected by drugs, environmental exposures, and nutritional or cofactor defects. In patients with LHON, smoking and excessive alcohol consumption have been reported to influence the course of disease (Kirkman et al., 2009), and the use of aminoglycosides may increase the risk of deafness in m.1555A>G mutation carriers (Nguyen and Jeyakumar, 2019). Whether these risk factors are associated with m.3243A>G mutation-related diseases have not been confirmed. Furthermore, many m.3243A>G mutation-related diseases are influenced by age, which is strongly associated with the severity of diabetes, hearing impairment, cerebellar ataxia, and neuropathy, confounding analysis (Pickett et al., 2018).

## Genetic counseling

The diversity of m.3243A>G presentations makes genetic counseling and choosing reproductive options extremely challenging. During recent decades procedures have been optimized to prevent the inheritance of mtDNA genetic diseases; prenatal diagnosis (PND), PGD, MRT and oocyte donation. PND is used to predict the risk of the fetus being severely affected after birth by measuring heteroplasmy of the fetus, most commonly using chorionic villus sampling (CVS) and amniotic fluid samples (AFS) (Smeets et al., 2015). However, the reliability of CVS is questionable, as a biopsy does not represent the heteroplasmy of fetuses in later pregnancy. Meanwhile, AFS has been proven to have similar heteroplasmy levels with that of the umbilical cord blood after birth (Steffann et al., 2021). At present, fetal heteroplasmy levels below 30% are generally defined as low risk, and those above 60% are defined as high risk. Steffann et al. examined heteroplasmy in fetal AFS or CVS from 51 mitochondrial mutation carriers (including 26 carriers of m.3243A>G) and found that the transmission risk of heteroplasmy from mother to fetus was 73%, including 21 fetuses with <30% heteroplasmy, 21 with 30%–60% heteroplasmy, and 15 with >60% heteroplasmy. The placenta as a whole appears to be a reliable reflection of the heteroplasmy of the fetus, but the heteroplasmy of the placenta is very unevenly distributed (Vachin et al., 2018). PND is less costly and may be appropriate for parents who have a low risk of recurrence. However, due to the high variability of phenotypes and the lack of large amounts of data, there is an intermediate zone of heteroplasmy that would present an unknown phenotype, making it difficult for patients to decide to continue or terminate a pregnancy.

PGD utilizes heteroplasmy measurements from one or several cells of pre-implantation embryos. A healthy embryo with no or very low mutation load can then be selected for embryo transfer (Sallevelt et al., 2017). In most cases, blastomere biopsy of the cleavage embryo appears to be representative of the entire embryo, and for m.3243A>G, a single-blastomere biopsy is sufficient to make a reliable diagnosis of PGD (Sallevelt et al., 2017). Although polar body (PB) biopsy is feasible and practical, studies have shown that the heteroplasmy determined in the PB does not accurately reflect that of the embryo (Neupane et al., 2014). Trophectoderm biopsy could allow biopsy of more cells, and of cells that do not contribute to the developing fetus thus making it potentially less damaging to embryo development than blastomere biopsy, but its accuracy is not confirmed (Treff et al., 2012; Neupane et al., 2014). In PGD, a key consideration is the threshold of heteroplasmy with which to identify safe embryos. Recently studies suggested a cutoff of 18% heteroplasmy for PGD was safe for most mtDNA mutations, although m.3243A>G was an exception where a lower level (<15%) may be needed (Hellebrekers et al., 2012; Sallevelt et al., 2017).

If all the embryos produced by female carriers have high mutation loads, as is more likely for women carrying high levels of mutation load themselves, PGD cannot help and MRT must be considered. However, MRT is not approved for clinical use in most countries, and more clinical trials are needed to confirm its safety and efficacy (Wang et al., 2014). Treatment with donor eggs can completely block the inheritance of deleterious mutations, but it still faces ethical and legal challenges in some countries (Poulton et al., 2017).

Overall, heteroplasmy levels are the key factor in genetic counseling. The analysis of heteroplasmy must be thorough as different tissues may harbor variable heteroplasmy levels. Heteroplasmy levels should also be adjusted for age for accurate interpretation. Importantly, Rebolledo found that the mother’s age of conception was positively associated with elevated mutation load in a child (Rebolledo-Jaramillo et al., 2014), likely attributable to oocyte ageing. Thus, the age of a patient must be considered. No matter which procedures are available to patients, wider factors should be taken into account in genetic counseling. As age increases, mature oocytes might carry higher mutation loads and ovarian function might decrease, potentially compromising chances of success in PGD and MRT. One study has found a higher number of mtDNA mutations in the progeny of older pregnant women (Rebolledo-Jaramillo et al., 2014). Since nuclear genetic factors are difficult to determine precisely, the incidences of disease among family members could also be taken into account. Finally, studies have shown that mtDNA copy number is associated with heteroplasmy level and phenotype (Grady et al., 2018), and so it is recommended to measure mtDNA copy number in muscle biopsies before making clinical decisions.

## Conclusion

In this review, we summarize the distinctive clinical phenotypic and genetic characteristics of m.3243A>G mitochondrial disease. In the past few years, the characteristics of the m.3243A>G mutation have been greatly elucidated. Heteroplasmy at mtDNA site 3243 might be more common than previously thought. Both drift and selection seem to affect m.3243A>G heteroplasmy over time. In addition, elucidating the role of nuclear gene expression provides additional information about the pathways that m.3243A>G affects, and reveals new entry points for understanding the pathological effects of diseases. Finally, we detailed the genetic interventions currently available to clinicians to provide positive reproductive options for families with m.3243A>G mitochondrial disease.

Extensive and in-depth studies are still needed in the near future. Future work must reveal the exact contribution of drift and selection to shifting heteroplasmy in available models. Further elucidation of disease mechanisms is also necessary to understand why specific patients have tissue-specific phenotypes in mitochondrial disease and guide the development of novel therapies.
